# Using structural equation modelling to understand the contributors to anaemia among young Burkinabe children

**DOI:** 10.1111/mcn.12881

**Published:** 2019-09-03

**Authors:** Lilia Bliznashka, Joanne E. Arsenault, Elodie Becquey, Marie T. Ruel, Deanna K. Olney

**Affiliations:** ^1^ Poverty, Health and Nutrition Division International Food Policy Research Institute (IFPRI) Washington DC; ^2^ Department of Global Health and Population Harvard T.H. Chan School of Public Health Boston Massachusetts; ^3^ Program in International and Community Nutrition University of California Davis California USA

**Keywords:** anaemia, Burkina Faso, children, determinants of anaemia, iron deficiency

## Abstract

Anaemia is a persistent problem among young Burkinabe children, yet population‐specific information on its determinants is scant. We used baseline data from an evaluation of Helen Keller International's Enhanced Homestead Food Production Program (*n*=1210 children) to quantify household‐, mother‐, and child‐level factors associated with anaemia in Burkinabe children aged 6‐12 months. We used structural equation modelling to assess a theoretical model, which tested four categories of factors: (a) household food security and dietary diversity, (b) household sanitation and hygiene (latrine and poultry access and bednet ownership), (c) maternal factors (anaemia, stress, cleanliness, and health, hygiene and feeding knowledge and practices), and (d) child nutrition and health (iron deficiency (ID), retinol binding protein (RBP), malaria, and inflammation). The model also included household socio‐economic status, size, and polygamy; maternal age and education; and child age and sex. Results showed that ID, malaria, and inflammation were the primary direct determinants of anaemia, contributing 15%, 10%, and 10%, respectively. Maternal knowledge directly explained improved child feeding practices and household bednet ownership. Household dietary diversity directly explained 18% of child feeding practices. Additionally, RBP, child age and sex, and maternal anaemia directly predicted child haemoglobin. Our findings suggest that program effectiveness could be increased by addressing the multiple, context‐specific contributors of child anaemia. For young Burkinabe children, anaemia control programs that include interventions to reduce ID, malaria, and inflammation should be tested. Other potential intervention entry points suggested by our model include improving maternal knowledge of optimal health, hygiene, and nutrition practices and household dietary diversity.

**Key messages**

- Child anaemia is a persistent global problem, with a complex aetiology necessitating the understanding of context‐specific contributors to inform appropriate intervention design.
- This analysis showed that ID, malaria, and inflammation were the primary direct contributors to anaemia among young Burkinabe children.
- Our theoretical model based on the *Lancet* framework for optimal fetal and child nutrition and development suggests that other potential intervention entry points include improving maternal knowledge of optimal health, hygiene, and nutrition practices and improving household dietary diversity.
- Child anaemia control programs can increase their effectiveness by understanding and addressing the multiple, context‐specific contributors of anaemia.

## 1 INTRODUCTION

Child anaemia is a severe public health concern, affecting 43% of children 6‐59 months of age globally and 63% in Sub‐Saharan Africa in 2011 (World Health Organization, 2015). Anaemia, and iron deficiency anaemia in particular, has been linked to deficits in cognitive (Grantham‐McGregor & Ani, 2001; Hurtado, Claussen, & Scott, 1999; Sachdev, Gera, & Nestel, 2005; United Nations University, World Health Organization, Food and Agriculture Organization of the United Nations, & FAO, 2004; Walker et al., 2007; World Health Organization, 2001), motor (Grantham‐McGregor & Ani, 2001; Sachdev et al., 2005; Walker et al., 2007; World Health Organization, 2001), and socio‐emotional development (Grantham‐McGregor & Ani, 2001; United Nations University et al., 2004; Walker et al., 2007; World Health Organization, 2001), as well as learning and memory deficits in childhood and adulthood (Fretham, Carlson, & Georgieff, 2011; World Health Organization, 2001). These consequences of anaemia are associated with childhood and adolescent academic performance (Grantham‐McGregor & Ani, 2001; Walker et al., 2007; World Health Organization, 2001). Additionally, anaemia has been shown to negatively affect the immune system (Ekiz, Agaoglu, Karakas, Gurel, & Yalcin, 2005; Oppenheimer, 2001; World Health Organization, 2001) and reduce physical work performance (Haas & Brownlie, 2001; World Health Organization, 2001) and earning capacity (Horton & Ross, 2003).

In Burkina Faso, the setting for the present study, anaemia among preschool children is nearly universal, affecting 86% of children in 2016 (World Health Organization, 2017), and has shown little improvement since 2003 when the prevalence was 92% (Institut National de la Statistique et de la Démographie [INSD] et ICF International, 2012). Despite the severity of the problem, and the lack of progress in eliminating it, little is known about the factors determining anaemia among young Burkinabe children. To our knowledge, only two studies have looked at this issue among preschool children in this population. One assessed the non‐dietary predictors of anaemia among 61 orphans and vulnerable children 1‐6 years of age and found infection, diarrhoea, and stunting as the main contributors to anaemia (Sanou, O'Brien, & Desrosiers, 2008). The second study examined the geographical risk profile of anaemia among children 1‐4 years of age in Burkina Faso, Ghana, and Mali and identified severe malnutrition, malaria, and helminth infections as the main correlates of child anaemia in this area (Magalhaes & Clements, 2011).

With respect to interventions aimed at reducing child anaemia in Burkina Faso, small‐quantity lipid‐based nutrient supplements (SQ‐LNS) have been effective (Abbeddou et al., 2017) as has a nutrition‐sensitive agricultural intervention that included strong nutrition and health behaviour change communication (BCC) and women's empowerment strategies (Olney, Pedehombga, Ruel, & Dillon, 2015). However, these types of interventions only achieved reductions of 12‐18 percentage points, leaving the prevalence of child anaemia at approximately 75% among program beneficiaries. These results suggest that to effectively reduce the prevalence of child anaemia in Burkina Faso, programs should aim to comprehensively address the multiple determinants of anaemia based on a solid understanding of their importance and relative contribution to anaemia in a given context, rather than focus on single interventions such as improving micronutrient status, for example.

Although iron deficiency (ID) is recognized as the primary cause of anaemia worldwide and has been estimated to account for approximately 50% of child anaemia globally (World Health Organization, 2001), its contribution differs across settings. A recent study showed that the proportion of anaemia associated with ID among preschool children in West Africa varied between 5.0% in Sierra Leone and 35.9% in Liberia (Petry et al., 2016). Therefore, we expected the contribution of ID to anaemia among Burkinabe children to also be below the 50% global estimate. As the contribution of ID to anaemia varies across settings, so does the overall aetiology of anaemia. For example, factors such as malaria infection may play a larger role in explaining anaemia in some settings, whereas dietary factors may be more important in others. Studies have highlighted the contribution of several household‐, mother‐, and child‐level factors to child anaemia in different contexts. These include household use of unsafe drinking water (Ngnie‐Teta, Receveur, & Kuate‐Defo, 2007); maternal anaemia (Alaofè, Burney, Naylor, & Taren, 2017), and suboptimal infant and young child feeding (IYCF) practices (Alaofè et al., 2017; Woldie, Kebede, & Tariku, 2015); and child subclinical inflammation (Rawat et al., 2014), malaria infection (Calis et al., 2008; Magalhaes & Clements, 2011; Righetti et al., 2012), wasting (Calis et al., 2008, underweight (Rawat et al., 2014), and micronutrient deficiencies (Calis et al., 2008; Pasricha et al., 2010; Rawat et al., 2014). In Burkina Faso up to 70% of children 6‐12 months tested positive for malaria in 2010/2011, only 3% met minimum dietary diversity, 18% and 9% had consumed vitamin A‐ or iron‐rich foods in the past 24 hr, and only 14% lived in households with access to improved latrines (Institut National de la Statistique et de la Démographie [INSD] et ICF International, 2012). In addition, a study in Burkina Faso showed that 33% of children aged 9‐18 months had subclinical inflammation in 2010 (Abbeddou et al., 2017). Thus, we hypothesized that many of these factors played a role in child anaemia in Burkina Faso, as well as other factors outlined in the *Lancet* framework for optimal fetal and child nutrition and development (Black et al., 2013). Additional factors related to child anaemia include child age and sex (Alaofè et al., 2017; Cornet et al., 1998; Diouf et al., 2015; Ngnie‐Teta et al., 2007; Rawat et al., 2014; Semedo, Santos, Baião, Luiz, & da Veiga, 2014; Siegel et al., 2006; Woldie et al., 2015), maternal age (Alaofè et al., 2017; Semedo et al., 2014) and education (Calis et al., 2008; Diouf et al., 2015; Woldie et al., 2015), and household socio‐economic status (SES; Ngnie‐Teta et al., 2007; Semedo et al., 2014; Siegel et al., 2006; Woldie et al., 2015).

Given the excessively high prevalence of child anaemia in Burkina Faso, the scarcity of evidence on its determinants, the absence of progress in tackling the problem, and the limited effectiveness of interventions tested in this context, our objectives were twofold: (a) to understand the contemporary profile of anaemia among children 6‐12 months of age in Burkina Faso and (2) to examine the relative contributions of different household‐, maternal‐, and child‐level factors to child anaemia and the interrelations among them. Our aim was to identify the main modifiable factors contributing to child anaemia in this setting to help guide the design of programs and policies to tackle the severe problem of anaemia in this population.

## 2 METHODS

### 2.1 Study setting

This study took place in Gourma province in the Eastern region of Burkina Faso. The primary economic activities among men and women in the region were subsistence farming, and sales and services. Child malnutrition was one of the main problems in the region, characterized by inadequate dietary intake in terms of both quantity and quality of foods (Institut National de la Statistique et de la Démographie [INSD] et ICF International, 2012). Stunting, wasting, anaemia, and malaria were prevalent among children <5 years (Institut National de la Statistique et de la Démographie [INSD] et ICF International, 2012). Polygamy was common, with 52% of women in the Eastern region living in a polygamous union (Institut National de la Statistique et de la Démographie [INSD] et ICF International, 2012).

### 2.2 Data used

We used baseline data from a longitudinal cluster‐randomized trial of the Creating Homestead Agriculture for Nutrition and Gender Equity (CHANGE) program implemented by Helen Keller International (HKI) from 2014 to 2016 in 60 villages in four departments in Gourma province. CHANGE was a nutrition‐ and gender‐sensitive agriculture program with a strong nutrition, health, and hygiene BCC strategy targeted to households with children <12 months of age. A total of 2,494 households, corresponding to all eligible households with children <12 months of age, participated in the baseline survey conducted between February and May 2014. We used a sample of 1,210 children 6‐12 months of age to describe the contemporary aetiology of child anaemia prior to the start of this nutrition‐sensitive agriculture program designed to improve children's nutritional status, including anaemia. Ethical approval was received from the Ministry of Health of Burkina Faso and the institutional review board of IFPRI. The trial was registered with http://ClinicalTrials.gov, number NCT02236468.

### 2.3 Measurements

Household surveys collected data on household, maternal, and child socio‐economic and demographic characteristics and factors related to child anaemia. At the household level, food security, dietary diversity, and water, sanitation and hygiene (WASH) infrastructure were measured. Maternal factors assessed included stress and health, nutrition, hygiene and malaria knowledge and practices. Finally, data on child morbidity in the prior two weeks were also collected.

Clinical assessments were conducted for maternal and child haemoglobin (Hb) and additional biomarkers for children. Hb was assessed immediately from a finger or heel capillary blood sample using HemoCue® 201+ (Ängelholm, Sweden). Among children, malaria infection was assessed immediately using a rapid diagnostic test (RDT; Standard Diagnostics, Bioline Malaria Rapid Diagnostic Test). An additional 300 ul of capillary blood was collected in prelabelled microvettes and later used to assess serum ferritin (SF), soluble transferrin receptor (sTfR), retinol binding protein (RBP), C‐reactive protein (CRP), and α‐1 acid glycoprotein (AGP). Blood samples were kept in an ice box maintained at 1‐8° C until transport to the central laboratory at the district's hospital at the end of each day. Upon arrival, the blood samples were centrifuged for serum separation and 100 ul of serum were aliquoted for analysis. The samples were temporarily stored in a freezer at ‐22° C in the central laboratory until shipment on dry ice to VitA‐Fe Tech laboratories (Willstatt, Germany) where the micronutrient and inflammation marker analyses were conducted.

### 2.4 Theoretical model

The theoretical model proposed in this article builds on the *Lancet* framework for optimal fetal and child nutrition and development (Black et al., 2013). Household‐, maternal‐, and child‐level factors that we expected to affect child Hb concentration and anaemia were grouped in the following four categories: (a) household food security and dietary diversity (i.e., food security and use of food); (b) household sanitation and hygiene (i.e., access to a safe and hygienic environment); (c) maternal factors, including nutrition, stress, knowledge and practices related to child health and nutrition (i.e., feeding resources, breastfeeding and eating routine, feeding practices), and (d) child nutrition and health (i.e., low burden of infectious disease)**.**

Child anaemia was defined as Hb < 11 g/dl (World Health Organization, 2011a). Detailed descriptions of the factors included in the model are provided in Table S1 in the supporting information. Briefly:

1. *Household food security and dietary diversity*. Household food security was assessed using the food secure category from the Household Food Insecurity Access Scale (HFIAS; Coates, Swindale, & Bilinsky, 2007). Household dietary diversity was assessed by calculating a household dietary diversity score from a 7‐day recall of food consumption (Swindale & Bilinsky, 2006).
2. *Household sanitation and hygiene*. Sanitation was measured using an indicator of whether the household had access to latrines. Hygiene was assessed using an indicator of whether the child had access to poultry directly or to the part of the compound where poultry spent the most time. Bednet ownership was defined as a household owning at least one bednet. Initially, household handwashing, water storage and treatment, and garbage storage and disposal practices were considered in this category (United States Agency for International Development, n.d.). However, due to limited evidence on a link between household WASH practices and child anaemia (based on correlation analyses) and the lack of variability in our sample, these WASH practices were excluded from the final model.
3. *Maternal factors*. Maternal nutritional status was represented by whether or not she was anaemic (Hb<11 g/dl in pregnant women and women with missing pregnancy status and Hb<12 g/dl in non‐pregnant women (World Health Organization, 2011a)). Maternal stress was measured using the Self‐Reported Questionnaire 20 (SRQ; Beusenberg et al., 1994). Maternal knowledge of optimal health, hygiene, nutrition, and malaria practices was assessed using a summary score of correct answers to a set of 17 questions (possible range 0‐17). The topics in the knowledge score included breastfeeding (three questions), identification of vitamin A‐ and iron‐rich foods (two summary indicators), complementary feeding (two questions), feeding sick and recovering children (six questions), critical handwashing moments (one summary indicator), malaria symptoms (one summary indicator), causes of malaria (one question), and ways to prevent malaria (one summary indicator). Maternal hygiene practices were assessed using spot‐check observations of maternal and child cleanliness (Ruel & Arimond, 2002); an indicator was constructed for whether or not both the mother and child were all visibly clean according to the enumerator observation (hands, hair, face, and clothes). Additionally, maternal IYCF practices were assessed using the World Health Organization (WHO) indicators for consumption of iron‐rich foods and meeting a minimum acceptable diet (MAD) in the past 24 hr (World Health Organization et al., 2010), and an indicator for early introduction of complementary foods, before 6 months of age.
4. *Child nutritional and health status*. ID and RBP were used as indicators of child nutritional status. Given the high prevalence of inflammation in our sample (56%), ID was defined as sTfR >8.3 mg/l because sTfR is not as sensitive to inflammation as SF is (Erhardt, Estes, Pfeiffer, Biesalski, & Craft, 2004). RBP was adjusted for the presence of inflammation (Larson et al., 2017) and included as a continuous factor due to its limitations in assessing individual vitamin A deficiency (World Health Organization, 2011b) and the lack of a standard RBP cut‐off for defining vitamin A deficiency (Whitehead et al., 2015). Child health included malaria and subclinical inflammation. Malaria was defined as a positive RDT. The presence of subclinical inflammation was defined as CRP >5 mg/l (World Health Organization, 2014) or AGP >1 g/l (Thurnham, Mburu, Mwaniki, & Wagt, 2005). This combined definition allowed us to detect both recently infected children (with elevated CRP) and recovering children in later stages of inflammation (with elevated AGP; Thurnham & Mccabe, 2010). Biologically implausible values for SF, sTfR, RBP, CRP, and AGP were removed during the laboratory assessment following standard quality protocols. Although child diarrhoea is a known predictor of child anaemia (Ngnie‐Teta et al., 2007; Semedo et al., 2014; Woldie et al., 2015), it was not included in the model for two reasons. First, the household surveys measured diarrhoea period prevalence, accounting only for disease burden rather than duration and severity, which are likely more important contributors to child anaemia. Second, the household surveys aimed at collecting information on maternal health‐seeking behaviour with respect to symptoms of childhood illness, rather than information on the severity of the symptoms themselves.

All these factors were considered modifiable immediate determinants of child anaemia. In addition, the model also included known modifiable underlying factors associated with child anaemia, namely, mother's education, and household SES, size, and polygamy, as well as nonmodifiable factors, specifically child age and sex, and mother's age. Mother's education was defined as having any formal schooling. Household SES was assessed using a housing quality index, constructed using principal components analysis (Filmer & Pritchett, 2001).

The same model was tested replacing child anaemia with Hb concentration as the outcome variable.

### 2.5 Statistical analysis

**Figure 1:**
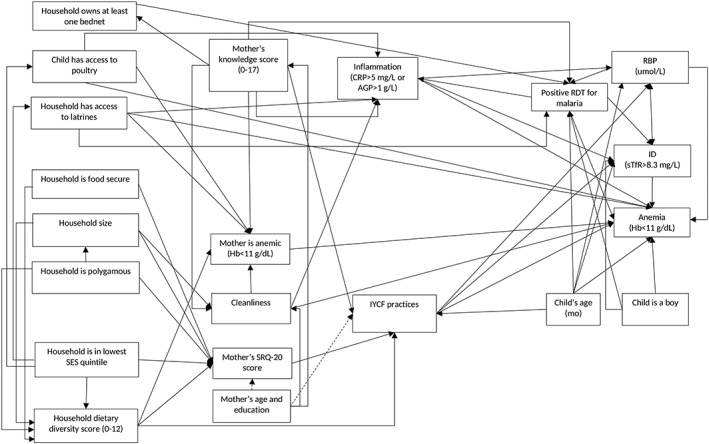
Conceptual model of the child‐, mother‐ and household‐level factors predicting anaemia among children 6‐12 months of age. Mother's anaemia was defined as Hb concentration <11 g/dl in pregnant mothers and mothers with missing pregnancy status and <12 g/dl in non‐pregnant mothers. Abbreviations used: Hb: haemoglobin; ID: iron deficiency; sTfR: soluble transferrin receptor; RBP: retinol binding protein; RDT: rapid diagnostic test; CRP: C‐reactive protein; AGP: α‐1‐acid glycoprotein; SRQ: self‐reported questionnaire; SES: socio‐economic status. Not depicted in the diagram are disturbance terms and their variances, the variances of exogenous variables, the covariances between exogenous variables, the scaling constant for the direct paths from disturbance terms to endogenous variables, and the covariance between the disturbance terms of “RBP (umol/l)” and “ID (sTfR>9.3 mg/l)”

To describe all the hypothesized relations between the household‐, mother‐, and child‐level factors associated with child anaemia, we developed a conceptual path diagram (Figure 1). We used structural equation modelling (SEM) to simultaneously estimate the 57 linear relations identified in the path diagram (Kline, 2015; StataCorp, 2017b). We present standardized coefficients from simultaneous linear regressions of the factors directly associated with anaemia. These are equivalent to standardized direct effects (SDE). Additionally, to examine the full set of direct and indirect relations in the theoretical model, we calculated standardized indirect effects (SIE) and standardized total effects (STE). SIEs are mediating effects, with multiple pathways added together into one indirect effect (Kline, 2015). For a specific path, SDE and SIE add to STE. The sum of the STEs represents the proportion of anaemia explained by the factors included in the model. Together with the proportion of anaemia not explained by the model, STEs add up to 1. This allows us to make inferences about the contribution of each factor to the prevalence of anaemia in this population. Thus, the STE of an individual factor can be interpreted as the proportion of anaemia explained by this factor, and the sum of all STEs can be interpreted as the proportion of anaemia explained by the model, and the difference between the sum of all STEs and 1 as the proportion of anaemia not explained by the model (Kline, 2015; StataCorp, 2017b).

Bivariate correlations of all factors included in the model are shown in Table S2. Endogenous factors were formally tested for multivariate normality (Doornik & Hansen, 2008). Given the sensitivity of such tests to small deviations of normality, the distributions of the continuous factors were also examined visually to determine whether they approximated a normal distribution. No transformations of the factors were employed. To account for missing values on any of the endogenous factors included in the model, we used a full information maximum likelihood estimator. To account for multiple comparisons, *p*‐values were corrected using the Benjamini–Hochberg correction controlling the false discovery rate (Benjamini & Hochberg, 1995), which is recommended in SEM (Cribbie, 2007). Standardized direct, indirect, and total effects were considered statistically significant if the *p*‐value was less than the corrected overall critical *p*‐value of .007 for total effects, .014 for direct effects, and .002 for indirect effects. All tests were two‐sided. Standard errors were adjusted for clustering at the village level, accounting for the original study design. All statistical analyses were conducted using STATA 15 (StataCorp, 2017a).

To test the robustness of the model to non‐normality, we refit the model using a robust maximum likelihood estimator in Mplus Version 8.2 (Muthén & Muthén, 2017). Results presented in Table S3 showed that our estimates for Hb concentration were identical, and our estimates for child anaemia were in the same direction and generally of a similar magnitude and statistical significance. Therefore, we determined that non‐normality was not likely to bias our model estimates. Subsequently, we presented results for the child anaemia and Hb concentration models fit using the full information maximum likelihood procedure in Stata, which were more easily interpretable for both outcomes.

The final analysis sample included 1,210 children. With 98 free parameters to estimate, we had a ratio of 13 observations to 1 linear relations, meeting the minimum requirement of 10 observations per linear relation commonly applied when using SEM (Tanaka, 1987). We estimated a model with 59 degrees of freedom.

To evaluate the overall fit of the model, we used the coefficient of determination (CD), which was the only fit statistic available after accounting for clustering and missing data. CD is a measure of fit equivalent to R‐squared. Applied to the current analyses, CD represents the proportion of variance in anaemia explained by all the factors included in the theoretical model, or how well the data fits around the regression lines. A higher value is preferred, with no explicit thresholds indicating poor model fit.

## 3 RESULTS

### 3.1 Sample characteristics

Children had a mean age of 8.9 mo (standard deviation (SD) 1.8), and 50% were boys (Table 1). Prevalence of child anaemia, ID, malaria, and inflammation were high. Maternal‐level factors showed high prevalence of anaemia, suboptimal feeding practices, low formal education, and low knowledge scores. However, cleanliness was satisfactory. Children lived in large households, many of which were polygamous. There was a high prevalence of food insecurity, low household dietary diversity, and poor sanitation and hygiene in our sample.

**Table 1:** Child, mother, and household characteristics of children 6‐12 months of age

Indicator	Mean ± *SD* or Percent
*N*	1,210
Child characteristics	
Age (months)	8.90±1.77
Boys (%)	50.17%
Hb concentration (g/dl)	9.43±1.29
Anemic (Hb concentration <11 g/dl)	88.23%
Adjusted SF (ug/L)	25.28±27.56
sTfR (mg/L)	14.15±5.98
Iron deficiency (sTfR >8.3 mg/L)	89.33%
Adjusted RBP (retinol equivalents umol/L)	0.96±0.23
Positive RDT for malaria	19.85%
CRP (mg/L)	7.02±12.43
Elevated CRP (CRP >5 mg/L)	30.99%
AGP (g/L)	1.10±0.43
Elevated AGP (AGP >1)	51.74%
Inflammation (CRP>5 mg/L or AGP>1 g/L)	55.72%
Child was fed minimum acceptable diet	10.40%
Child was introduced early to complementary foods	56.73%
Child consumed iron‐rich foods in past 24 hr	28.60%
Mother characteristics	
Age (years)	28.47±6.57
Any formal education	18.35%
Hb concentration (g/dl)	11.93±1.36
Anemic (Hb concentration <11 g/dl)^a^	47.90%
SRQ‐20 score	3.58±3.86
Knowledge score (0‐17)	7.95±2.81
Cleanliness	73.65%
Household characteristics	
Food secure	23.97%
Dietary diversity score (0‐12)	6.32±2.40
Has access to latrines	13.47%
Child has access to poultry	79.59%
Owns at least one bednet	96.78%
Lowest SES quintile	24.21%
Polygamous	41.57%
Number of children <6 years	3.09±1.62

Abbreviations: AGP: α‐1‐acid glycoprotein; CRP: C‐reactive protein; Hb: haemoglobin; RBP: retinol binding protein; RDT: rapid diagnostic test; SES: socio‐economic status; SF: serum ferritin; SRQ: self‐reported questionnaire; sTfR: soluble transferrin receptor.

a Anaemia was defined as Hb concentration <11 g/dl in pregnant mothers and mothers with missing pregnancy status and <12 g/dl in non‐pregnant mothers.

### 3.2 Model fit

Model fit was similar for both the child anaemia and Hb models: CD of 0.48 and 0.49, respectively.

### 3.3 Primary factors predicting child anaemia

**Figure 2:**
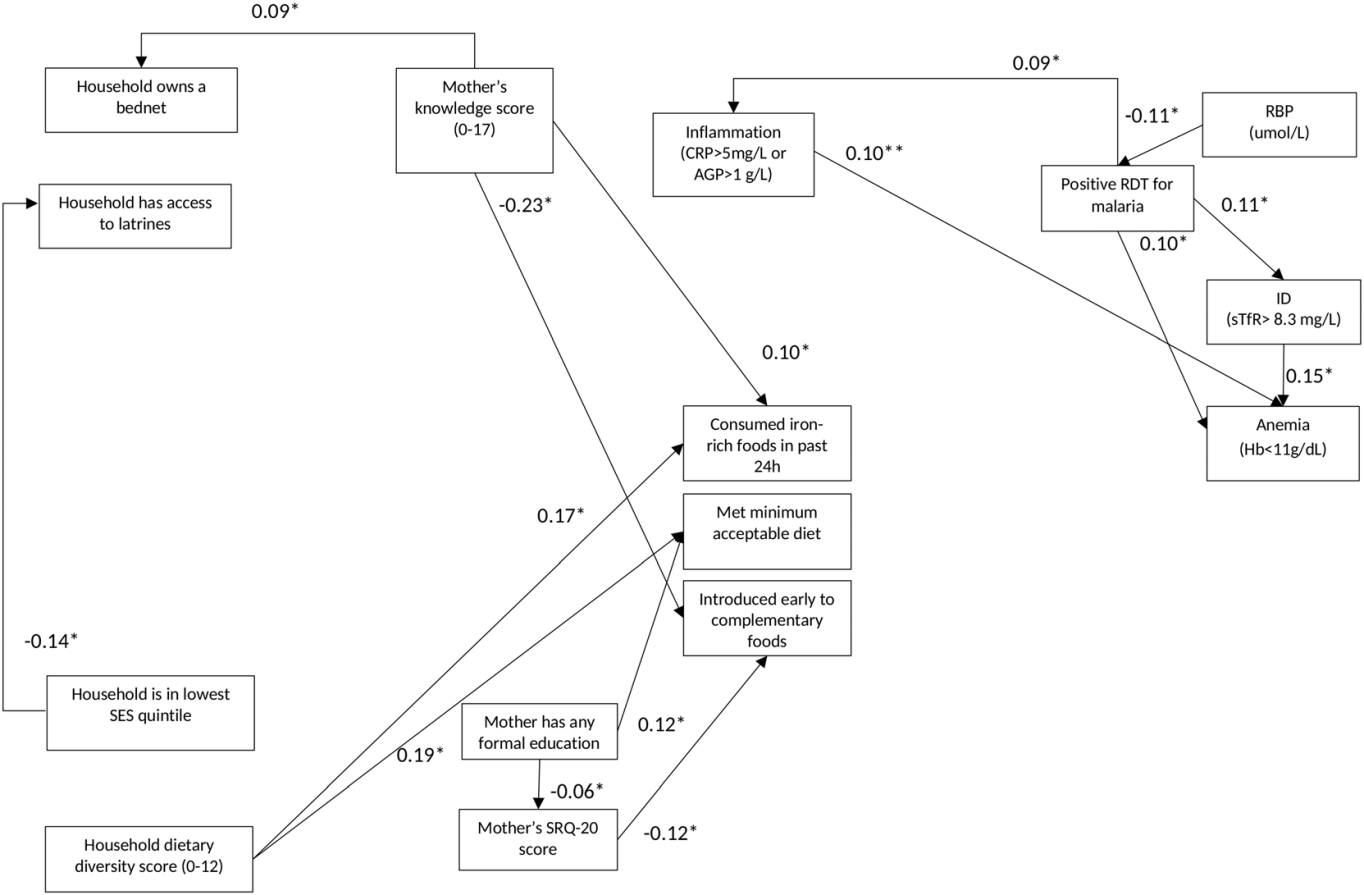
Direct effects of child‐, mother‐, and household‐level factors predicting anaemia among children 6‐12 months of age along the hypothesized pathways. All values are standardized direct effects (SDE). *P*‐values were corrected for multiple comparisons. Significance level: *p<.014. Abbreviations used: Hb: haemoglobin; ID: iron deficiency; sTfR: soluble transferrin receptor; RBP: retinol binding protein; RDT: rapid diagnostic test; CRP: C‐reactive protein; AGP: α‐1‐acid glycoprotein; SRQ: self‐reported questionnaire; SES: socio‐economic status. Direct effects of child age and sex were not included in the figure. Direct effects of non‐modifiable factors were not included in the figure.

The sum of the STEs was 0.68; thus, the model explained 68% of child anaemia. The primary child‐level predictors were ID (SDE 0.15, *p*=.005), malaria (SDE 0.10, *p*=.009), and inflammation (SDE 0.10, *p*=.012; Table 2 and Figure 2). The results were robust to an alternative ID definition using SF only (SF<12ug/L). Using this definition, the estimated direct contribution of ID to anaemia was 15% (results not shown). Similarly, in the model with Hb concentration as the outcome variable, ID (SDE ‐0.20, *p*<.001), malaria (SDE ‐0.19, *p*=.005), and inflammation (SDE ‐0.15, *p*=.004) were the primary child‐level predictors. In addition, RBP (SDE 0.08, *p*=.015), child age (SDE ‐0.12, *p*=.013) and sex (SDE ‐0.14, *p*=.006), and mother's anaemia (SDE ‐0.09, *p*=.014) also predicted child Hb concentration.

**Table 2:** Standardized total, direct, and indirect effects of the child‐, mother‐, and household‐level factors predicting anaemia and haemoglobin concentration among children 6‐12 months of age^a^

	Anaemia model			Hb model		
	Type of effect Standardized coefficient ± SE			Type of effect Standardized coefficient ± SE		
Structural relation	Total	Direct	Indirect	Total	Direct	Indirect
Child factors						
ID (sTfR >8.3 mg/l)	0.15±0.03*	0.15±0.03*	—	‐0.23±0.03*	‐0.20±0.02*	—
Adjusted RBP (retinol equivalents umol/L)	‐0.03±0.03	‐0.02±0.03	‐0.01±0.00*	0.09±0.03*	0.08±0.02*	0.02±0.01
Positive RDT for malaria	0.12±0.03*	0.10±0.03*	0.03±0.01*	‐0.23±0.04*	‐0.19±0.03*	‐0.04±0.01*
Inflammation (CRP >5 mg/l or AGP >1g/l)	0.10±0.03*	0.10±0.03*	0.00±0.01	‐0.15±0.02*	‐0.15±0.02*	0.00±0.01
Child age (mo)	0.09±0.03*	0.06±0.03	0.03±0.01	‐0.17±0.03*	‐0.12±0.04*	‐0.05±0.01*
Child is a boy	0.08±0.03	0.07±0.03	0.01±0.01	‐0.16±0.03*	‐0.14±0.03*	‐0.02±0.01
Mother factors						
Mother is anaemic (Hb concentration <11 g/dl)^b^	0.06±0.03	0.06±0.03	—	‐0.09±0.03*	‐0.09±0.03*	—
Mother's SRQ‐20 score	‐0.01±0.00	—	‐0.01±0.00	0.01±0.00	—	0.01±0.00
Cleanliness	0.01±0.03	0.01±0.03	0.00±0.00	‐0.02±0.03	‐0.01±0.03	‐0.00±0.01
Child fed minimum acceptable diet	0.01±0.03	0.00±0.03	0.01±0.00	‐0.00±0.03	0.00±0.03	‐0.01±0.01
Child consumed iron‐rich foods in past 24 hr	0.02±0.03	0.03±0.03	‐0.01±0.01	‐0.03±0.03	‐0.04±0.03	0.01±0.01
Child introduced early to complementary foods	0.05±0.03	0.05±0.03	—	‐0.05±0.03	‐0.05±0.03	—
Mother's knowledge score (0‐17)	‐0.02±0.01	—	‐0.02±0.01	0.03±0.01	—	0.03±0.01
Mother's age (years)	0.00±0.00	—	0.00±0.00	0.00±0.00	—	0.00±0.00
Mother has any formal education	0.01±0.00	—	0.01±0.00	‐0.01±0.01	—	‐0.01±0.01
Household factors						
Household is food secure	0.00±0.00	—	0.00±0.00	‐0.00±0.00	—	‐0.00±0.00
Household dietary diversity (0‐12)	0.01±0.01	—	0.01±0.01	‐0.01±0.01	—	‐0.01±0.01
Household has access to latrines	‐0.00±0.02	0.00±0.02	‐0.01±0.00	0.03±0.02	0.01±0.02	0.02±0.01
Child has access to poultry	0.05±0.03	0.05±0.03	0.00±0.00	‐0.01±0.03	‐0.01±0.03	‐0.00±0.01
Household owns at least one bednet	‐0.00±0.00	—	‐0.00±0.00	0.01±0.01	—	0.01±0.01
Household is in lowest SES quintile	0.00±0.00	—	0.00±0.00	‐0.00±0.00	—	‐0.00±0.00
Household is polygamous	‐0.00±0.00	—	‐0.00±0.00	0.00±0.00	—	0.00±0.00
Number of children <6 years	0.00±0.00	—	0.00±0.00	‐0.00±0.00	—	‐0.00±0.00

Abbreviations: AGP: α‐1‐acid glycoprotein; CRP: C‐reactive protein; Hb: haemoglobin; ID: iron deficiency; RBP: retinol binding protein; RDT: rapid diagnostic test; SES: socio‐economic status; SRQ: self‐reported questionnaire; sTfR: soluble transferrin receptor.

a SEs were adjusted for clustering. *P*‐values were corrected for multiple comparisons.

b Anaemia was defined as Hb concentration <11 g/dl in pregnant mothers and mothers with missing pregnancy status and <12 g/dl in non‐pregnant mothers.

* Significance level: *p*<.007 for total effects, *p*<.014 for direct effects, and *p*<.002 for indirect effects.

### 3.4 Categories of factors explaining child anaemia

No factors in the household food security and dietary diversity category predicted child anaemia directly. However, higher dietary diversity score was directly linked to improved feeding practices: a higher proportion of children meeting MAD or consuming iron‐rich foods (SDE 0.19, *p*=.003 and SDE 0.17, *p*=.003, respectively; Table 3 and Figure 2).

None of the factors in the sanitation and hygiene category directly or indirectly explained child anaemia. Access to latrines was directly associated with lower prevalence of malaria, but these results were only marginally significant (SDE ‐0.05, *p*=.020).

Similarly, none of the maternal factors were associated with child anaemia directly or indirectly. However, higher maternal knowledge was directly associated with a higher proportion of children consuming iron‐rich foods in the past 24 hr (SDE 0.10, *p*<.010), a lower proportion of children introduced early to complementary foods (SED ‐0.23, *p*=.002), and a higher likelihood of the household owning a bednet (SDE 0.09, *p*=.014). Surprisingly, higher maternal stress was associated with a lower proportion of children introduced early to complementary foods (SDE ‐0.12, *p*=.008).

Child ID directly predicted higher anaemia prevalence (SDE 0.15, *p*=.005) and RBP had a small indirect effect on anaemia of SIE ‐0.01, *p*<.002, through malaria. In addition, malaria (SDE 0.10, *p*=.009) and inflammation (SDE 0.10, *p*=.012) significantly predicted higher prevalence of child anaemia (Table 2 and Figure 2). Malaria predicted higher child anaemia prevalence directly (SDE 0.10, *p*=.009) as well as indirectly (SIE 0.03, *p*<.001) through its direct effect on ID and inflammation.

Among the modifiable underlying factors considered, mother's education and household SES were directly associated with immediate determinants of child anaemia. Mother's education directly predicted a higher proportion of children meeting MAD (SDE 0.12, *p*=.007), and low household SES was directly associated with lower access to latrines (SDE ‐0.14, *p*=.008; Table 3). Finally, from the non‐modifiable factors included in the model, child older age predicted child anaemia (STE 0.09, *p*=.007) and maternal age was positively associated with knowledge (SDE 0.10, *p*=.010).

**Table 3:** Standardized total, direct, and indirect effects of the child‐, mother‐, and household‐level factors predicting the categories of factors in the anaemia model^a^

	Anaemia model		
	Type of effect Standardized coefficient ± SE		
Structural relation	Total	Direct	Indirect
Sanitation and hygiene category			
Select factors predicting household access to latrines			
Household is in lowest SES quintile	‐0.14±0.04*	‐0.14±0.04*	—
Select factors predicting household bednet ownership			
Mother's knowledge score (0‐17)	0.09±0.03	0.09±0.03*	—
Maternal factors category			
Select factors predicting child being fed a minimum acceptable diet			
Mother has any formal education	0.12±0.03*	0.12±0.03*	0.00±0.00
Household dietary diversity score (0‐12)	0.19±0.03*	0.19±0.03*	‐0.00±0.00
Select factors predicting child consumption of iron‐rich foods			
Mother's knowledge score (0‐17)	0.10±0.03*	0.10±0.03*	—
Household dietary diversity score (0‐12)	0.17±0.03*	0.17±0.03*	0.00±0.00
Select factors predicting child being introduced early to complementary foods			
Mother's SRQ 20	‐0.12±0.03*	‐0.12±0.03*	—
Mother's knowledge score (0‐17)	‐0.23±0.03*	‐0.23±0.03*	—
Child nutrition and health category			
Select factors predicting ID			
Adjusted RBP (retinol equivalents umol/L)	‐0.01±0.00*	—	‐0.01±0.00*
Positive RDT for malaria	0.11±0.02*	0.11±0.02*	0.00±0.00
Select factors predicting child malaria			
Adjusted RBP (Retinol Equivalents umol/L)	‐0.11±0.03*	‐0.11±0.03*	0.00±0.00
Select factors predicting inflammation			
Positive RDT for malaria	0.09±0.03	0.09±0.03*	‐0.00±0.00

Abbreviations: AGP: α‐1‐acid glycoprotein; CRP: C‐reactive protein; Hb: haemoglobin; ID: iron deficiency; RBP: retinol binding protein; RDT: rapid diagnostic test; SES: socio‐economic status; SRQ: self‐reported questionnaire; sTfR: soluble transferrin receptor.

a SEs were adjusted for clustering. *P*‐values were corrected for multiple comparisons.

* Significance level: *p*<.007 for total effects, *p*<.014 for direct effects, and *p*<.002 for indirect effects.

### 3.5 Factors predicting child Hb concentration

Similar results were seen for Hb concentration. The only differences in the significant relations between the anaemia and Hb models were that in the Hb model child RBP, older age, and male sex directly predicted Hb concentration (SDE 0.08, *p*=.014, SDE ‐0.12, *p*=.013, and SDE ‐0.14, *p*=0.006, respectively), and mother's anaemia directly predicted lower Hb concentration (SDE ‐0.09, *p*=.014; Table 2).

## 4 DISCUSSION

Our study found that, of the factors assessed, child ID, malaria, and inflammation were the three main contributors to child anaemia among 6‐12‐month old Burkinabe children. ID was the main contributor, explaining 15% of child anaemia in this population. This proportion was in line with a recent study indicating a lower relative contribution of ID to anaemia in countries with high prevalence of anaemia and inflammation (Petry et al., 2016). These findings indicate that although targeting ID alone will likely decrease the prevalence of ID, it may only lead to modest reductions in the prevalence of child anaemia. Of the modifiable factors assessed, our study also found that malaria infection and inflammation each explained 10% of child anaemia prevalence. In turn, inflammation was predicted by malaria infection, itself a modifiable factor. These findings highlight the importance of measuring factors other than ID when assessing the aetiology of child anaemia in a particular context to design successful interventions intended to reduce anaemia.

This is the first study to use SEM to examine a theoretical model of the relative contributions of different household‐, mother‐, and child‐level factors to anaemia among young Burkinabe children and the ways in which they are related to each other. Our findings indicated that model fit was acceptable, and in this population the hypothesized factors were associated with child anaemia along the proposed categories of factors. This type of modelling approach allowed us to simultaneously estimate all the direct and indirect relations among all the factors included in the model and to portion out the direct and indirect contributions of all modifiable and non‐modifiable factors, accounting for the complex, multifactorial nature of anaemia. SEM and similar approaches may therefore be more appropriate than multivariate regression for estimating the relative contributions of different factors to anaemia and thus can aid in understanding its multifaceted aetiology from cross‐sectional studies.

In addition to identifying the primary contributors to child anaemia, using SEM allowed to identify some entry points for interventions to reduce anaemia among young Burkinabe children. For example, with respect to the household sanitation and hygiene category of factors, increasing access to latrines can help reduce the burden of malaria and, in turn, reduce inflammation, ID and child anaemia. Although we found no direct associations between WASH practices (e.g., handwashing, water storage and treatment, garbage collection, and disposal) and child anaemia, improving water and sanitation practices can help reduce child anaemia indirectly by reducing the prevalence of malaria by limiting mosquito breeding sites within the household. However, this mechanism was not formally tested in our model. It is important to note that the current study was conducted among young children 6‐12 months of age during the dry season and the prevalence of malaria in our sample was 20%, which was much lower than the estimated 46% prevalence among preschoolers during the peak transmission season (Institut National de la Statistique et de la Démographie [INSD], Programme National de Lutte contre le Paludisme,, & ICF International, 2015). Therefore, our findings likely represent a lower bound of the contribution of malaria to child anaemia. In addition, improving WASH practices can potentially help reduce child morbidity, which in turn could decrease inflammation. The lack of association between WASH practices and child anaemia in the current analyses should be interpreted with caution as it could be due to the low prevalence of optimal WASH practices, e.g. only 1% of households in our sample had a handwashing station and <1% of households used appropriate methods for storing and disposing of garbage.

Although the IYCF practices assessed were not directly associated with ID or child anaemia, our results do highlight the potential role of mothers' knowledge and household dietary diversity in improving IYCF practices. While household diet did not predict child anaemia, it was positively and significantly associated with better diets of children as indicated by the proportion of children meeting MAD and consuming iron‐rich foods. Likewise, mothers' knowledge was directly associated with the latter of these child diet indicators. The lack of association between these IYCF practices and child anaemia is similar to that found in a study in Bangladesh where the authors found that meeting MAD and consuming iron‐rich foods did not directly predict child anaemia (Rawat et al., 2014). Similarly, a recent study in Burkina Faso found no relation between iron intake and iron status among preschool children (Martin‐Prevel et al., 2016). These findings in our sample are not surprising. Iron status, which could be improved by more optimal IYCF practices, is only one of the key factors associated with anaemia in our sample. Additionally, the lack of direct association between meeting MAD and consuming iron‐rich foods in our sample may also be due to the potentially small quantities of food consumed by young children and the commonly consumed phytate‐rich, cereal‐based diets (Arsenault et al., 2014). The relations between children's diets and ID and anaemia are complex and not fully understood, especially with regard to bioavailability of iron in contexts such as Burkina Faso with unvaried, phytate‐rich diets, poor WASH conditions, and high prevalence of inflammation and malaria.

Mothers' knowledge was associated with a variety of optimal IYCF, care, and health practices. However, none of these individual practices were directly associated with child anaemia in our analyses. Nevertheless, these findings underscore the potential of improving mothers' knowledge of health, hygiene, nutrition, and malaria practices as a way to affect multiple mother‐ and household‐level factors that can improve children's general wellbeing and living environment, and health and nutritional status. Well‐designed BCC strategies can be particularly effective in this setting. First, they have been shown to improve mother's knowledge and adoption of optimal health, hygiene and feeding practices in multiple settings (Hoddinott, Ahmed, Karachiwalla, & Roy, 2017; Kim et al., 2016; Nair et al., 2017; Saaka, Aryee, Kuganab‐Lem, Ali, & Masahudu, 2017), including Burkina Faso (Olney et al., 2015; Sarrassat et al., 2015). Second, they can be tailored to specific age groups and settings and thus can be designed to address the known determinants of child anaemia in a given population. For instance, given the high prevalence of malaria in this part of Burkina Faso, and its importance as a contributor to child anaemia, ID, and inflammation, BCC strategies should include modules on prevention, symptoms, and treatment of malaria among the most vulnerable household members.

Unexpectedly, maternal stress was associated with a lower proportion of children being introduced early to complementary foods. It is possible that stress as perceived by Burkinabe women may not be captured well by the SRQ‐20. Although widely used, to our knowledge, the SRQ‐20 has not been validated for use among mothers of young children in Burkina Faso.

Among the last group of factors related to child nutrition and health, our results indicate that interventions and programs should aim to reduce ID, which was the main contributor to child anaemia in this population, reduce malaria and inflammation, and improve RBP, which was associated with higher child Hb concentration and lower prevalence of malaria. A combination of interventions can help achieve this. For example, SQ‐LNS when given alongside active disease surveillance has been shown to effectively improve child micronutrient status and to reduce anaemia among young Burkinabe children (Abbeddou et al., 2017). A nutrition‐sensitive agriculture program, providing agricultural training and inputs and a health, hygiene, and nutrition BCC intervention, in Eastern Burkina Faso has also been shown to reduce child anaemia (Olney et al., 2015), demonstrating how multisectoral programs can work to reduce anaemia by modifying multiple factors.

Despite the many strengths of this study, including the use of SEM and the ample data on child‐, mother‐, and household‐level factors along the hypothesized categories of anaemia determinants, there are several limitations. First, we lacked data on sickle cell disease and beta thalassemia, which are common genetic disorders in Burkina Faso (Devoucoux et al., 1991; Grosse et al., 2011; Kafando, Sawadogo, Cotton, Vertongen, & Gulbis, 2005; Piel et al., 2013) known to contribute to anaemia. Second, although we had data on child‐, mother‐, and household‐level dietary diversity, we lacked data on the amount of foods eaten and the preparation methods, which would have helped understand the statistically insignificant relation between child anaemia and consumption of iron‐rich foods. Third, apart from iron and vitamin A status, we had no data on other micronutrient deficiencies, which have been shown to cause anaemia, such as vitamin B12 or folate deficiency (Scott, 2007), and which are likely highly prevalent among mothers and children in the region (Rohner et al., 2013; Shahab‐Ferdows et al., 2015). Given that 32% of anaemia remained unexplained by our model, further studies should assess the contribution of these additional factors to child anaemia in this context. Finally, the cross‐sectional nature of the study did not allow us to establish causality. The planned subsequent longitudinal analysis of the CHANGE program, which aimed to modify multiple factors in the theoretical model, will help us understand the extent to which the main contributors to child anaemia in this population were modifiable over time in this setting.

## 5 CONCLUSION

We used SEM to estimate a theoretical model of the modifiable and non‐modifiable household‐, mother‐, and child‐level factors hypothesized to predict anaemia among children 6‐12 months of age in Burkina Faso. Our results indicated that, of the factors assessed, ID, malaria, and inflammation were the main contributors to child anaemia in the study population, directly explaining 15%, 10%, and 10%, respectively. These findings showed that the contribution of ID was lower than typically expected, highlighting the importance of understanding the multifaceted aetiology of child anaemia when designing anaemia control programs. This approach allowed us to identify multiple factors that could be tackled to reduce anaemia in infants in Burkina Faso including improving children's iron and vitamin A status; improving mothers' health, hygiene and malaria prevention knowledge and practices; and improving the health sector capacity to identify and treat malaria in young children. Our findings confirmed that iron supplementation can only go so far in addressing anaemia in countries like Burkina Faso where anaemia is caused by multiple modifiable environmental and behavioural nutrition, health and sanitation factors. In these contexts, holistic approaches to understanding and tackling the multiple factors responsible for child anaemia are urgently needed.

## CONFLICTS OF INTEREST

The authors declare that they have no conflicts of interest.

## CONTRIBUTIONS

LB and DKO conceptualized the analyses presented in this paper. DKO, EB, and MR designed the CHANGE program evaluation, and EB and DKO led the data collection activities. LB, DKO, and JEA led the data analyses. LB and DKO drafted the manuscript. All authors had full access to the data and contributed to interpreting and discussing the results and revising the manuscript. All authors read and approved the final version of the paper. LB had final responsibility for submitting this article for publication.

## Supporting information


**Table S1.** Description of household‐, mother‐ and child‐level variables included in the model^a^
**Table S2.** Bivariate correlations of the child‐, mother‐ and household‐level factors predicting anemia and hemoglobin concentration among children 6‐12 mo ^a^
**Table S3.** Unstandardized total, direct and indirect effects of the child‐, mother‐ and household‐level factors predicting anemia and hemoglobin concentration among children 6‐12 mo of age estimated using full information maximum likelihood and robust maximum likelihood^a^
**Table S4.** Standardized direct effects for child‐, mother‐ and household‐level factors predicting anemia and hemoglobin concentration among children 6‐12 mo of age ^a^
