# Supplementary material for: Using structural equation modelling to understand the contributors to anaemia among young Burkinabe children
Source: Matern Child Nutr. 2019 Sep 3;16(1):e12881. doi: 10.1111/mcn.12881 (PMC7038887; doi:10.1111/mcn.12881)
Supplement: Supplementary file 1 — Table S1. Description of household‐, mother‐ and child‐level variables included in the modela Table S2. Bivariate correlations of the child‐, mother‐ and household‐level factors predicting anemia and hemoglobin concentration among children 6‐12 mo a Table S3. Unstandardized total, direct and indirect effects of the child‐, mother‐ and household‐level factors predicting anemia and hemoglobin concentration among children 6‐12 mo of age estimated using full information maximum likelihood and robust maximum likelihooda Table S4. Standardized direct effects for child‐, mother‐ and household‐level factors predicting anemia and hemoglobin concentration among children 6‐12 mo of age a [file MCN-16-e12881-s001.docx]

**Supplemental Table 1** Description of household-, mother- and child-level variables included in the model^a^

| **Factor** | **Description** | **Variable code and properties** | **Reference** |
| --- | --- | --- | --- |
| *Household-level* | | | |
| Food security | Assessed using the Household Food Insecurity Access Scale (HFIAS), which consists of four categories: (1) food secure, (2) mildly food insecure, (3) moderately food insecure, and (4) severely food insecure | Binary indicator:  1 = Household is food secure (HFIAS = 1)  0 = Household is not food secure (HFIAS = 2 or HFIAS = 3 or HFIAS = 4)  Cronbach’s alpha for study sample = 0.73 | Coates, J., Swindale, A., & Bilinsky, P. (2007). *Household Food Insecurity Access Scale (HFIAS) for measurement of food access: indicator guide*. Washington D.C. |
| Household dietary diversity | Assessed using the Household Dietary Diversity Score | Count variable, ranging from 0 to 12  Cronbach’s alpha for study sample = 0.81 | Swindale, A., & Bilinsky, P. (2006). *Household Dietary Diversity Score (HDDS) for Measurement of Household Food Access: Indicator Guide (v.2)*. *Washington, DC: Food and Nutrition Technical …*. Washington D.C. |
| Hygiene | Assessed using an indicator of whether the child had access to poultry directly or to the part of the compound where poultry spent most of the time | Binary indicator:  1 = Yes, child has access  0 = No, child does not have access |  |
| Sanitation | Assessed using an indicator of whether the household had access to latrines | Binary indicator:  1 = Yes, household has access to latrines  0 = No, household does not have access to latrines |  |
| Bednet ownership | Assessed using an indicator for whether the household owned at least one bednet | Binary indicator:  1 = Households owns ≥ 1 bednets  0 = Household owns 0 bendets |  |
| Household size | Assessed based on household head report of the number of children <6 y living in the household | Count variable, ranging from 1 to 9 |  |
| Household polygamy | Based on household head report of polygamy | Binary indicator:  1 = Household is polygamous  0 = Household is monogamous |  |
| Household is in the lowest socio-economic quintile | Assessed using a housing quality index, constructed using principal components analysis (PCA). The PCA was conducted using 4 variables floor material (house had a non-dirt floor), roof material (house had a non-straw roof), walls material (house had cement walls), and availability of electricity. The continuous index score was divided into 5 equal quintiles. | Binary indicator:  1 = Household is in the lowest quintile of the housing quality index  0 = Household is not in the lowest quintile of the housing quality index (i.e. household is in the second, third, fourth or fifth quintile of the household quality index) | Filmer, D., & Pritchett, L. H. (2001). Estimating Wealth Effects Without Expenditure Data — or Tears: An Application to Educational Enrollments in States of India. *Demography*, *38*(1), 115–132. https://doi.org/10.1353/dem.2001.0003 |
| *Mother-level* | | | |
| Mother’s nutritional status | Based on clinical assessment of hemoglobin (Hb) from finger or heel capillary blood sample using Hemocue.  Hb concentrations >90 g/dL were recoded to missing as they are considered biologically implausible values. | Binary indicator:  1 = Mother is anemic (Hb <11 g/dL if woman is pregnant or pregnancy status is unknown, or Hb<12 g/dL if woman is not pregnant)    0 = Mother is not anemic (Hb≥11 g/dL if woman is pregnant or pregnancy status is unknown, or Hb≥12 g/dL if woman is not pregnant) | World Health Organization. (2011). Haemoglobin concentrations for the diagnosis of anaemia and assessment of severity. Vitamin and Mineral Nutrition Information System. Geneva: WHO/NMH/NHD/MNM/11.1 |
| Mother’s stress | Assessed using the WHO Self-Reported Questionnaire (SRQ) | Count variable, ranging from 0 to 1  Cronbach’s alpha for study sample = 0.90 | World Health Organization. (1994). *A user’s guide to the self-reporting questionnaire (SRQ)*. Geneva. |
| Mother’s knowledge score | Assessed using a summary score of correct answers to a set of 17 questions:   - Breastfeeding (n=3 questions) - Identification of vitamin A and iron-rich foods (n=2 summary indicators for whether the mother correctly identified at least two vitamin-A rich foods, and both liver and meat/fish as iron-rich foods, respectively) - Complementary feeding (n=2 questions) - Feeding sick and recovering children (n=6 questions) - Critical handwashing moments (n=1 summary indicator for whether the mother correctly identified all five critical handwashing moments) - Malaria symptoms (n=1 summary indicator for whether the mother correctly identified at least two malaria symptoms) - Causes of malaria (n=1 question for whether the mother identified mosquitoes as a cause of malaria) - Ways to prevent malaria (n=1 summary indicator for whether the mother correctly identified at least two ways to prevent malaria)   For each question, correct answers were coded as 1, incorrect answers were coded as 0, and “do not know” responses were replaced with missing. | Count variable, ranging from 0 to 17 |  |
| Mother’s hygiene practices | Assessed using a spot check observation of cleanliness. Mother and child hands, hair, face and clothes were assessed for whether they were clean, dusty or dirty. | Binary indicator:  1 = mother and child are both all visibly clean  0 = mother and/or child are not both all visibly clean | Ruel, M. T., & Arimond, M. (2002). Spot-check observational method for assessing hygiene practices: review of experience and implications for programmes. *Journal of Health, Population, and Nutrition*, *20*(1), 65–76. |
| Mother’s infant and young child feeding practices | Assessed using the WHO infant and young child feeding practices indicators for consumption of iron-rich foods and meeting a minimum acceptable diet. Assessment based on maternal recall in the past 24 h.  An additional indicator was created for early introduction of complementary foods before 6 mo of age. Assessment was based on maternal recall of when child was first fed complementary foods | Binary indicators:  Consumption of iron-rich foods:  1 = Child was fed iron-rich foods in the past 24 h  0 = Child was not fed iron-rich foods in the past 24 h  Minimum acceptable diet:  1 = Child met minimum acceptable diet in the past 24 h  0 = Child did not meet minimum acceptable diet in the past 24 h  Early introduction to complementary foods:  1 = Child was fed complementary foods before child turned 6 mo  0 = Child was not fed complementary foods before child turned 6 mo | World Health Organization, UNICEF, USAID, AED, UCDAVIS, & IFPRI. (2010). *Indicators for assessing infant and young child feeding practices. Part II Measurement*. |
| Mother’s age | Based on maternal report, expressed in years | Count variable, ranging from 16 to 66 |  |
| Mother’s education | Based on maternal report of completed education level | Binary indicator:  1 = Mother has any formal schooling  0 = Mother has no formal schooling |  |
| *Child-level* | | | |
| Iron deficiency | Assessed using a blood sample. Defined based on soluble transferring receptor (sTfR) which is not sensitive to inflammation | Binary indicator:  1 = sTfR >8.3 mg/l  0 = sTfR ≤8.3 mg/l | World Health Organization. (2011). Serum transferrin receptor levels for the assessment of iron status and iron deficiency in populations. Vitamin and Mineral Nutrition Information System. Geneva: WHO/NMH/NHD/MNM/14.6  Erhardt, J. G., Estes, J. E., Pfeiffer, C. M., Biesalski, H. K., & Craft, N. E. (2004). Combined Measurement of Ferritin, Soluble Transferrin Receptor, Retinol Binding Protein, and C-Reactive Protein by an Inexpensive, Sensitive, and Simple Sandwich Enzyme-Linked Immunosorbent Assay Technique. *The Journal of Nutrition*, *134*(11), 3127–3132. https://doi.org/10.1093/jn/134.11.3127 |
| Vitamin A status | Assessed using a blood sample, based on retinol binding protein (RBP) adjusted for the presence of inflammation | Continuous variable, units are Retinol Equivalents umol/L | World Health Organization. (2011). Serum retinol concentrations for determining the prevalence of vitamin A deficiency in populations. Geneva: WHO/NMH/NHD/MNM/11.3  Larson, L. M., Namaste, S. M., Williams, A. M., Engle-Stone, R., Addo, O. Y., Suchdev, P. S., … Northrop-Clewes, C. A. (2017). Adjusting retinol-binding protein concentrations for inflammation: Biomarkers Reflecting Inflammation and Nutritional Determinants of Anemia (BRINDA) project. *The American Journal of Clinical Nutrition*, *106*(Suppl 1), 390S–401S. https://doi.org/10.3945/ajcn.116.142166 |
| Malaria | Assessed using a rapid diagnostic test (RDT). | Binary indicator:  1 = Positive RDT  0 = Negative RDT |  |
| Inflammation | Assessed using a blood sample. Defined based C-reactive protein (CRP) and α-1 acid glycoprotein (AGP) | Binary indicator:  1 = CRP >5 mg/l or AGP >1 g/l  0 = CRP ≤5 mg/l and AGP ≤1 g/l | Thurnham, D. I., Mburu, A. S. W., Mwaniki, D. L., & Wagt, A. De. (2005). Micronutrients in childhood and the influence of subclinical inflammation. *Proceedings of the Nutrition Society*, *64*(04), 502–509. https://doi.org/10.1079/PNS2005468  World Health Organization. (2014). C-reactive protein concentrations as a marker of inflammation or infection for interpreting biomarkers of micronutrient status. Vitamin and Mineral Nutrition Information System. Geneva: WHO/NMH/NHD/EPG/14.7.  Thurnham, D. I., & Mccabe, G. P. (2010). Influence of infection and inflammation on biomarkers of nutritional status with an emphasis on vitamin A and iron. *Geneva, World Health Organization*, 15–17. |
| Child anemia | Based on clinical assessment of hemoglobin (Hb) from finger or heel capillary blood sample using Hemocue.  Hb concentrations >90 g/dL were recoded to missing as they are considered biologically implausible values. | Binary indicator:  1 = Child is anemic (Hb < 11 g/dL)  0 = Child is not anemic (Hb ≥ 11 g/dL) | World Health Organization. (2011). Haemoglobin concentrations for the diagnosis of anaemia and assessment of severity. Vitamin and Mineral Nutrition Information System. Geneva: WHO/NMH/NHD/MNM/11.1 |
| Child age | Age in months calculated as the difference between the date of interview and date of birth (reported by the mother) | Continuous, ranging from 6 to 12 months |  |
| Child sex |  | Binary indicator:  1 = Child is a boy  0 = Child is a girl |  |

^a^ Abbreviations used: AGP, α-1-acid glycoprotein; CRP, C-reactive protein; Hb, hemoglobin; HFIAS, Household Food Insecurity Access Scale; PCA, principal components analysis; RBP, retinol binding protein; RDT, rapid diagnostic test; SES, socio-economic status; SRQ, self-reported questionnaire; sTfR, soluble transferrin receptor.

**Supplemental Table 2** Bivariate correlations of the child-, mother- and household-level factors predicting anemia and hemoglobin concentration among children 6-12 mo ^a^

|  | 1 | 2 | 3 | 4 | 5 | 6 | 7 | 8 | 9 | 10 | 11 | 12 | 13 | 14 | 15 | 16 | 17 | 18 | 19 | 20 | 21 | 22 | 23 | 24 |
| --- | --- | --- | --- | --- | --- | --- | --- | --- | --- | --- | --- | --- | --- | --- | --- | --- | --- | --- | --- | --- | --- | --- | --- | --- |
| Child is anemic^b^ | − |  |  |  |  |  |  |  |  |  |  |  |  |  |  |  |  |  |  |  |  |  |  |  |
| Child is iron deficiencient^c^ | 0.18** | − |  |  |  |  |  |  |  |  |  |  |  |  |  |  |  |  |  |  |  |  |  |  |
| Child’s RBP | -0.02 | 0.05 | − |  |  |  |  |  |  |  |  |  |  |  |  |  |  |  |  |  |  |  |  |  |
| Child has positive RDT for malaria | 0.12** | 0.11** | -0.11** | − |  |  |  |  |  |  |  |  |  |  |  |  |  |  |  |  |  |  |  |  |
| Child inflammation^d^ | 0.12** | 0.03 | 0.02 | 0.09** | − |  |  |  |  |  |  |  |  |  |  |  |  |  |  |  |  |  |  |  |
| Child age | 0.1** | 0.13** | -0.01 | 0.08** | 0.07* | − |  |  |  |  |  |  |  |  |  |  |  |  |  |  |  |  |  |  |
| Child is a boy | 0.08** | 0.11** | -0.04 | -0.05 | 0.03 | 0.00 | − |  |  |  |  |  |  |  |  |  |  |  |  |  |  |  |  |  |
| Mother is anemic^e^ | 0.06* | 0.04 | 0.00 | -0.03 | 0.05 | 0.00 | -0.01 | − |  |  |  |  |  |  |  |  |  |  |  |  |  |  |  |  |
| Mother’s SRQ-20 score | -0.03 | 0.02 | 0.06* | -0.06 | 0.07* | 0.04 | -0.01 | 0.04 | − |  |  |  |  |  |  |  |  |  |  |  |  |  |  |  |
| Cleanliness | -0.02 | -0.04 | -0.01 | -0.08** | 0.01 | -0.11** | -0.02 | -0.03 | -0.04 | − |  |  |  |  |  |  |  |  |  |  |  |  |  |  |
| Child was fed MAD | 0.03 | 0.04 | 0.03 | 0.00 | 0.01 | 0.18** | -0.06* | -0.01 | 0.00 | -0.04 | − |  |  |  |  |  |  |  |  |  |  |  |  |  |
| Child was fed iron-rich foods | 0.05 | -0.01 | -0.01 | 0.04 | 0.00 | 0.26** | 0.01 | -0.02 | -0.02 | -0.05 | 0.39** | − |  |  |  |  |  |  |  |  |  |  |  |  |
| Child was introduced early to CF | 0.04 | -0.01 | -0.02 | -0.03 | 0.00 | -0.09** | 0.03 | -0.03 | -0.12** | -0.04 | 0.01 | -0.04 | − |  |  |  |  |  |  |  |  |  |  |  |
| Mother’s knowledge score | -0.02 | -0.05 | 0.03 | -0.06* | -0.06 | 0.00 | -0.02 | -0.04 | -0.03 | 0.07* | 0.08** | 0.11** | -0.23** | − |  |  |  |  |  |  |  |  |  |  |
| Mother’s age | -0.01 | -0.02 | 0.03 | 0.01 | -0.03 | -0.04 | -0.02 | 0.02 | 0.04 | 0.01 | -0.03 | 0.02 | -0.04 | 0.1** | − |  |  |  |  |  |  |  |  |  |
| Mother’s education | 0.01 | 0.03 | 0.05 | -0.05 | -0.02 | 0.04 | -0.01 | -0.03 | -0.02 | 0.07* | 0.13** | 0.06* | 0.05 | 0 | -0.05 | − |  |  |  |  |  |  |  |  |
| Household food security | 0.00 | -0.02 | 0.01 | 0.02 | 0.01 | 0.03 | -0.01 | 0.04 | -0.07* | 0.05 | 0.02 | -0.02 | -0.07* | 0.01 | -0.05 | 0.04 | − |  |  |  |  |  |  |  |
| Household dietary diversity | -0.01 | 0.01 | 0.04 | 0.03 | -0.04 | 0.03 | -0.02 | 0.01 | -0.02 | -0.09** | 0.2** | 0.18** | -0.03 | 0.04 | 0.04 | 0.00 | 0.08** | − |  |  |  |  |  |  |
| Household access to latrines | -0.01 | -0.02 | 0.02 | -0.06* | -0.02 | 0.05 | 0.01 | -0.03 | 0.04 | 0.03 | 0.02 | 0.03 | -0.09** | 0.11** | 0.03 | 0.06* | 0.08** | 0.07* | − |  |  |  |  |  |
| Child has access to poultry | 0.05 | -0.03 | 0.00 | 0.04 | 0.00 | 0.03 | 0.00 | 0.03 | 0.17** | -0.12** | 0.07* | 0.03 | -0.02 | -0.06 | 0.00 | 0.04 | 0.04 | 0.11** | 0.02 | − |  |  |  |  |
| Household owns a bednet | 0.01 | -0.03 | 0.04 | -0.04 | -0.02 | 0.03 | 0.01 | -0.02 | 0.01 | 0.02 | 0.01 | 0.03 | -0.07* | 0.09** | 0.05 | 0.01 | 0.06* | 0.02 | 0.02 | 0.02 | − |  |  |  |
| Household is in lowest SES quintile | 0.00 | -0.07* | -0.06* | -0.01 | -0.02 | -0.05 | 0.00 | -0.01 | 0.05 | -0.04 | -0.07* | -0.06* | 0.09** | -0.09** | -0.02 | -0.03 | -0.13** | -0.07* | -0.14** | 0.01 | 0.02 | − |  |  |
| Household is polygamous | 0.07* | 0.03 | 0.03 | 0.04 | 0.06* | -0.04 | -0.04 | 0.00 | -0.03 | 0.06 | -0.03 | -0.05 | -0.02 | -0.02 | 0.14** | -0.02 | 0.01 | -0.05 | 0.01 | -0.09** | -0.01 | -0.05 | − |  |
| Number of children <6 y | 0.01 | 0.01 | 0.02 | 0.06* | 0.04 | -0.05 | -0.02 | 0.03 | -0.03 | -0.01 | -0.04 | -0.03 | 0.00 | 0.01 | 0.25** | -0.05 | -0.02 | 0.03 | 0.00 | -0.06* | 0.04 | -0.04 | 0.58** | − |

^a^ All values are mean ± SD or percentages unless otherwise indicated. AGP, α-1-acid glycoprotein; CF, complementary food; CRP, C-reactive protein; Hb, hemoglobin; MAD, minimum acceptable diet; RBP, retinol binding protein; RDT, rapid diagnostic test; SES, socio-economic status; SRQ, self-reported questionnaire; sTfR, soluble transferrin receptor.

^b^ Defined as Hb concentration < 11 g/dL

^c^ Defined as sTfR >8.3 mg/L

^d^ Defined as CRP>5 mg/L or AGP>1 g/L

^e^ Defined as Hb concentration <11 g/dL in pregnant mothers and mothers with missing pregnancy status and <12 g/dL in non-pregnant mothers.

**Supplemental Table 3** Unstandardized total, direct and indirect effects of the child-, mother- and household-level factors predicting anemia and hemoglobin concentration among children 6-12 mo of age estimated using full information maximum likelihood and robust maximum likelihood^a^

|  | **Anemia** | | **Hb** | |
| --- | --- | --- | --- | --- |
| **Structural relationship** | **MLMV^b^** | **MLR^c^** | **MLMV^b^** | **MLR^c^** |
| *Child factors* |  |  |  |  |
| ID (sTfR >8.3 mg/l) | 0.15±0.03** | 2.80±0.59** | -0.84±0.09** | -0.84±0.09** |
| Adjusted RBP (Retinol Equivalents umol/L) | -0.02±0.04 | -0.23±0.41 | 0.42±0.14** | 0.42±0.14** |
| Positive RDT for malaria | 0.08±0.02** | 3.37±1.37 | -0.62±0.10** | -0.62±0.10** |
| Inflammation (CRP >5 mg/l or AGP >1g/l) | 0.06±0.02** | 1.87±0.36* | -0.39±0.06** | -0.39±0.06** |
| Child age (mo) | 0.01±0.01 | 0.11±0.06 | -0.09±0.03** | -0.09±0.03** |
| Child is a boy | 0.04±0.02* | 0.44±0.21* | -0.37±0.07** | -0.37±0.07** |
|  |  |  |  |  |
| *Mother factors* |  |  |  |  |
| Mother is anemic (Hb concentration <11 g/dL)^b^ | 0.04±0.02* | 1.43±0.24 | -0.23±0.07** | -0.23±0.07** |
| Cleanliness | 0.00±0.02 | 1.04±0.23 | -0.04±0.09 | -0.04±0.09 |
| Child fed minimum acceptable diet | 0.00±0.03 | 1.04±0.37 | 0.01±0.13 | 0.01±0.13 |
| Child consumed iron-rich foods in past 24h | 0.02±0.02 | 1.31±0.33 | -0.11±0.09 | -0.11±0.09 |
| Child introduced early to complementary foods | 0.03±0.02 | 1.42±0.28 | -0.12±0.07 | -0.12±0.07 |
|  |  |  |  |  |
| *Household factors* |  |  |  |  |
| Household has access to latrines | 0.00±0.02 | 1.02±0.23 | 0.04±0.06 | 0.04±0.06 |
| Child has access to poultry | 0.04±0.03 | 1.45±0.35 | -0.04±0.10 | -0.04±0.10 |

^a^ All values are unstandardized coefficients ± SE unless otherwise indicated. SEs were adjusted for clustering. P-values were not corrected for multiple comparisons. Significance level: *p<0.05, **p<0.01. Abbreviations used: Hb, hemoglobin; ID, iron deficiency; sTfR, soluble transferrin receptor; RBP, retinol binding protein; RDT, rapid diagnostic test; CRP, C-reactive protein; AGP, α-1-acid glycoprotein; SRQ, self-reported questionnaire; SES, socio-economic status.

^b^ Model was estimated using maximum likelihood missing values (MLMV) in Stata 15.

^c^ Model was estimated using robust maximum likelihood (MLR) in Mplus Version 8.2. Values are unstandardized coefficients ± SE or odds ratios (OR) ± SE in the anemia model.

**Supplemental Table 4** Standardized direct effects for child-, mother- and household-level factors predicting anemia and hemoglobin concentration among children 6-12 mo of age ^a^

|  | **Anemia model** | **Hb model** |
| --- | --- | --- |
| N | 1210 | 1210 |
| *Factors predicting anemia/Hb* |  |  |
| *Child factors* |  |  |
| ID (sTfR >8.3 mg/l) | 0.15±0.03* | -0.20±0.02* |
| Adjusted RBP (Retinol Equivalents umol/L) | -0.02±0.03 | 0.08±0.02* |
| Positive RDT for malaria | 0.10±0.03* | -0.19±0.03* |
| Inflammation (CRP >5 mg/l or AGP >1 g/l) | 0.10±0.03* | -0.15±0.02* |
| Child age (mo) | 0.06±0.03 | -0.12±0.04* |
| Child is a boy | 0.07±0.03 | -0.14±0.03* |
|  |  |  |
| *Mother factors* |  |  |
| Mother is anemic (Hb concentration <11 g/dL)^b^ | 0.06±0.03 | -0.09±0.03* |
| Child was fed minimum acceptable diet | 0.00±0.03 | 0.00±0.03 |
| Child consumed iron-rich foods in past 24h | 0.03±0.03 | -0.04±0.03 |
| Child was introduced early to complementary foods | 0.05±0.03 | -0.05±0.03 |
| Cleanliness | 0.01±0.03 | -0.01±0.03 |
|  |  |  |
| *Household factors* |  |  |
| Household has access to latrines | 0.00±0.02 | 0.01±0.02 |
| Child has access to poultry | 0.05±0.03 | -0.01±0.03 |
|  |  |  |
| *Factors predicting ID* |  |  |
| *Child factors* |  |  |
| Positive RDT for malaria | 0.11±0.02* | 0.11±0.02* |
| Inflammation (CRP >5 mg/l or AGP >1 g/l) | 0.01±0.04 | 0.01±0.04 |
| Child age (mo) | 0.13±0.03* | 0.13±0.03* |
| Child is a boy | 0.12±0.03* | 0.12±0.03* |
|  |  |  |
| *Mother factors* |  |  |
| Child was fed minimum acceptable diet | 0.05±0.03 | 0.05±0.03 |
| Child consumed iron-rich foods in past 24h | -0.05±0.03 | -0.05±0.03 |
|  |  |  |
| *Factors predicting RBP* |  |  |
| *Child factors* |  |  |
| Inflammation (CRP >5 mg/l or AGP >1 g/l) | 0.03±0.03 | 0.03±0.03 |
| Child age (mo) | -0.02±0.03 | -0.02±0.03 |
|  |  |  |
| *Mother factors* |  |  |
| Child was fed minimum acceptable diet | 0.03±0.03 | 0.03±0.03 |
|  |  |  |
| *Factors predicting child malaria* |  |  |
| *Child factors* |  |  |
| Adjusted RBP (Retinol Equivalents umol/L) | -0.11±0.02* | -0.11±0.02* |
| Child age (mo) | 0.09±0.03* | 0.09±0.03* |
| Child is a boy | -0.05±0.03 | -0.05±0.03 |
|  |  |  |
| *Mother factors* |  |  |
| Mother’s knowledge score (0-17) | -0.05±0.03 | -0.05±0.03 |
|  |  |  |
| *Household factors* |  |  |
| Household has access to latrines | -0.05±0.03 | -0.05±0.03 |
| Household owns at least one bednet | -0.03±0.03 | -0.03±0.03 |
|  |  |  |
| *Factors predicting inflammation* |  |  |
| *Child factors* |  |  |
| Positive RDT for malaria | 0.09±0.03* | 0.09±0.03* |
|  |  |  |
| *Mother factors* |  |  |
| Cleanliness | 0.02±0.03 | 0.02±0.03 |
| Mother’s knowledge score (0-17) | -0.05±0.03 | -0.05±0.03 |
|  |  |  |
| *Household factors* |  |  |
| Household has access to latrines | -0.01±0.03 | -0.01±0.03 |
| Child has access to poultry | -0.01±0.03 | -0.01±0.03 |
|  |  |  |
| *Factors predicting mother’s anemia* |  |  |
| *Mother factors* |  |  |
| Cleanliness | -0.02±0.03 | -0.02±0.03 |
| Mother’s knowledge score (0-17) | -0.03±0.03 | -0.03±0.03 |
|  |  |  |
| *Household factors* |  |  |
| Household dietary diversity score (0-12) | 0.01±0.03 | 0.01±0.03 |
| Household has access to latrines | -0.02±0.03 | -0.02±0.03 |
| Child has access to poultry | 0.02±0.03 | 0.02±0.03 |
|  |  |  |
| *Pathways to mother’s stress* |  |  |
| *Mother factors* |  |  |
| Mother’s age (y) | 0.04±0.03 | 0.04±0.03 |
| Mother has any formal education | -0.01±0.03 | -0.01±0.03 |
|  |  |  |
| *Household factors* |  |  |
| Household is food secure | -0.06±0.03 | -0.06±0.03 |
| Household dietary diversity score (0-12) | -0.02±0.04 | -0.02±0.04 |
| Household is in lowest SES quintile | 0.04±0.04 | 0.04±0.04 |
| Household is polygamous | -0.02±0.03 | -0.02±0.03 |
| Number of children <6y | -0.02±0.03 | -0.02±0.03 |
|  |  |  |
| *Factors predicting child being fed minimum acceptable diet* |  |  |
| *Child factors* |  |  |
| Child age (mo) | 0.17±0.03* | 0.17±0.03* |
|  |  |  |
| *Mother factors* |  |  |
| Mother’s SRQ-20 score | 0.01±0.03 | 0.01±0.03 |
| Mother’s knowledge score (0-17) | 0.08±0.03 | 0.08±0.03 |
| Mother’s age (y) | -0.04±0.02 | -0.04±0.02 |
| Mother has any formal education | 0.12±0.03* | 0.12±0.03* |
|  |  |  |
| *Household factors* |  |  |
| Household dietary diversity score (0-12) | 0.19±0.03* | 0.19±0.03* |
|  |  |  |
| *Factors predicting child consumption of iron-rich food* |  |  |
| *Child factors* |  |  |
| Child age (mo) | 0.26±0.03* | 0.26±0.03* |
|  |  |  |
| *Mother factors* |  |  |
| Mother’s SRQ-20 score | -0.02±0.03 | -0.02±0.03 |
| Mother’s knowledge score (0-17) | 0.10±0.03* | 0.10±0.03* |
| Mother’s age (y) | 0.02±0.03 | 0.02±0.03 |
| Mother has any formal education | 0.06±0.03 | 0.06±0.03 |
|  |  |  |
| *Household factors* |  |  |
| Household dietary diversity score (0-12) | 0.17±0.03* | 0.17±0.03* |
|  |  |  |
| *Factors predicting child being introduced early to complementary foods* |  |  |
| *Child factors* |  |  |
| Child age (mo) | -0.09±0.03* | -0.09±0.03* |
|  |  |  |
| *Mother factors* |  |  |
| Mother’s SRQ-20 score | -0.12±0.03* | -0.12±0.03* |
| Mother's knowledge score (0-17) | -0.23±0.03* | -0.23±0.03* |
| Mother’s age (y) | -0.01±0.03 | -0.01±0.03 |
| Mother has any formal education | 0.05±0.03 | 0.05±0.03 |
|  |  |  |
| *Factors predicting cleanliness* |  |  |
| *Mother factors* |  |  |
| Mother’s knowledge score (0-17) | 0.07±0.04 | 0.07±0.04 |
| Mother’s age (y) | 0.00±0.03 | 0.00±0.03 |
| Mother has any formal education | 0.07±0.03 | 0.07±0.03 |
|  |  |  |
| *Household factors* |  |  |
| Number of children <6 y | -0.00±0.03 | -0.00±0.03 |
|  |  |  |
| *Factors predicting mother’s knowledge* |  |  |
| Mother’s age (y) | 0.10±0.03* | 0.10±0.03* |
| Mother has any formal education | 0.00±0.03 | 0.00±0.03 |
|  |  |  |
| *Factors predicting household dietary diversity* |  |  |
| *Household factors* |  |  |
| Household is food secure | 0.08±0.06 | 0.08±0.06 |
| Household is in lowest SES quintile | -0.06±0.04 | -0.06±0.04 |
| Household is polygamous | -0.11±0.06 | -0.11±0.06 |
| Number of children <6 y | 0.09±0.05 | 0.09±0.05 |
|  |  |  |
| *Factors predicting household access to latrines* |  |  |
| *Household factors* |  |  |
| Household is in lowest SES quintile | -0.14±0.04* | -0.14±0.04* |
|  |  |  |
| *Factors predicting child access to poultry* |  |  |
| *Household factors* |  |  |
| Household is in lowest SES quintile | 0.01±0.03 | 0.01±0.03 |
|  |  |  |
| *Factors predicting household bednet ownership* |  |  |
| *Mother factors* |  |  |
| Mother's knowledge score (0-17) | 0.09±0.03* | 0.09±0.03* |
|  |  |  |
| *Household factors* |  |  |
| Household is in lowest SES quintile | 0.02±0.02 | 0.02±0.02 |
|  |  |  |
| *Factors predicting number of children <6y* |  |  |
| *Household factors* |  |  |
| Household is polygamous | 0.57±0.03* | 0.57±0.03* |
|  |  |  |
| CD^c^ | 0.48 | 0.49 |

^a^ All values are standardized coefficients ± SE unless otherwise indicated. SEs were adjusted for clustering. P-values were corrected for multiple comparisons. Significance level: *p<0.014. Hb, hemoglobin; ID, iron deficiency; sTfR, soluble transferrin receptor; RBP, retinol binding protein; RDT, rapid diagnostic test; CRP, C-reactive protein; AGP, α-1-acid glycoprotein; SRQ, self-reported questionnaire; SES, socio-economic status; CD, coefficient of determination.

^b^ Anemia was defined as Hb concentration <11 g/dL in pregnant mothers and mothers with missing pregnancy status and <12 g/dL in non-pregnant mothers.

^c^ CD is the only model fit index available with full information maximum likelihood and clustered SEs.
